# When donor T cells attack: The curious case of liver transplant-associated acute graft-versus-host-disease

**DOI:** 10.1177/00368504221117070

**Published:** 2022-08-17

**Authors:** Max Deschner, Donald J. Bastin, Ziad Solh, Karen Bosma, Wael Haddara, Ping Yang, Robert Broadbent, Aaron Haig, Jonathan Keow, Mayur Brahmania, Anargyros Xenocostas, Uday Deotare

**Affiliations:** 1 Department of Medicine, 6221 Western University, London, ON, Canada; 2 Division of Hematology, Department of Medicine, 6221 Western University, London, ON, Canada; 3 Department of Pathology and Laboratory Medicine, 6221 Western University, London, ON, Canada; 4 Division of Critical Care, Department of Medicine, 6221 Western University, London, ON, Canada; 5 Department of Pathology and Laboratory Medicine, 10033 London Health Sciences Centre, London, ON, Canada; 6 Division of Gastroenterology, Department of Medicine, 6221 Western University, London, ON, Canada

**Keywords:** Orthotopic liver transplantation, solid-organ transplant graft-versus-host disease, chimerism, cytogenetics

## Abstract

Graft versus host disease is a rare but deadly complication of solid organ transplant. Clinical features of graft-versus-host-disease are non-specific, which may lead to delayed diagnosis as more common conditions including infections or drug reactions are considered. We describe a 54-year-old male patient who underwent liver transplantation for alcohol use disorder-related cirrhosis and developed acute graft-versus-host disease. Initial clinical presentation included dermatitis, bone marrow failure and enteritis. Results of skin biopsy and cytogenetic studies were consistent with liver transplant-associated acute graft-versus-host disease. The importance of this case is to highlight to transplant physicians and surgeons the challenges of diagnosing graft-versus-host-disease. In our case, pre-existing partnerships among the liver and hematopoietic stem cell transplant teams, transfusion medicine specialists, critical care specialists and facilitated timely communication relevant to confirming graft-versus-host disease. We propose an algorithm to assist in the workup of suspected graft-versus-host disease. Because this condition is characterized by high mortality, a high index of suspicion is imperative for prompt diagnosis and optimal management of the donor-recipient immune interaction when patients present with classic clinical features.

## Introduction

Acute graft-versus-host disease (GVHD) is a rare but deadly complication of liver transplantation. Symptoms including fever, skin rash and diarrhea, laboratory evidence of liver dysfunction and bone marrow failure are non-specific, which can delay the diagnosis of GVHD. We describe a case of liver transplant-associated GVHD. We discuss challenges in the diagnosis of GVHD and argue that early involvement of hematopoietic stem cell transplant (HSCT) physicians can facilitate both timely identification and treatment of GVHD. Because this condition is characterized by high mortality, a high index of suspicion is imperative for early diagnosis and optimal management of the donor-recipient immune interaction when patients present with classic features.

## Case report

A 54-year-old male underwent liver transplantation in our tertiary referral centre for alcohol use disorder-related cirrhosis. The patient's blood type was A RhD positive. He received a deceased male donor transplant (donation after circulatory death) with blood type A RhD positive. The donor and recipient were a two-antigen match when considering A*, B*, C*, DRB1*, DRB345* and DQB1* loci (matches on DRB1* and DQB1* loci). His antirejection medications included basiliximab due to kidney dysfunction, and methylprednisolone followed by a prednisone taper, mycophenolate mofetil and tacrolimus. His postoperative hospital course was unremarkable.

On day 35 following transplantation, the patient was admitted to the hospital for a change in his mental status. In the hospital, the patient deteriorated rapidly. Three days after admission, he was transferred to the Intensive Care Unit with hypotension, hypothermia, anemia (hemoglobin 67 g/L, WBC count 3.6 × 10^9^/L, platelets 295 × 10^9^/L at time of transfer to the ICU) and a maculopapular rash. Given his nonspecific symptoms, he was empirically treated with ciprofloxacin and metronidazole for possible intraabdominal infection and acyclovir for possible viral meningitis. A lumbar puncture was performed and was positive for CMV in the CSF, however, this was a bloody tap and the patient had known CMV viremia for which he received treatment. Subsequent lumbar punctures were negative but it is unclear whether he had ever been truly positive in the CSF. Two days later, he was intubated for tachypnea and decreased level of consciousness. The patient did undergo an HRCT and showed no underlying infection. However, it did show bilateral minimal pleural effusions and associated atelectasis.

He developed pancytopenia, suspected to be secondary to ganciclovir versus cytomegalovirus infection (white blood cells <0.5 × 10^9^/L, platelets 16 × 10^9^/L and hemoglobin 75 g/L). He received non-irradiated blood as he did not meet indications for blood product irradiation. The patient was initially treated with Ganciclovir and then later changed to Foscarnet due to further risk of bone marrow suppression. He developed diarrhea and a bullous rash on the chest and arms but sparing the palms and soles (Days 58 and 61 post-transplant, respectively). He had a positive Nikolsky sign on skin examination. Total bilirubin was elevated at 30.9 μmol/L (peaking at 122.2 μmol/L), but alkaline phosphatase was normal. Differential diagnosis included toxic epidermal necrolysis, Stevens-Johnson Syndrome, transfusion-associated graft-versus-host-disease and liver transplant-associated graft-versus-host disease. Due to suspicion of transfusion-associated graft-versus-host-disease, he received irradiated cellular blood products thereafter. The HSCT team was then consulted to throw more light on the symptoms of pancytopenia.

**Figure 1.**
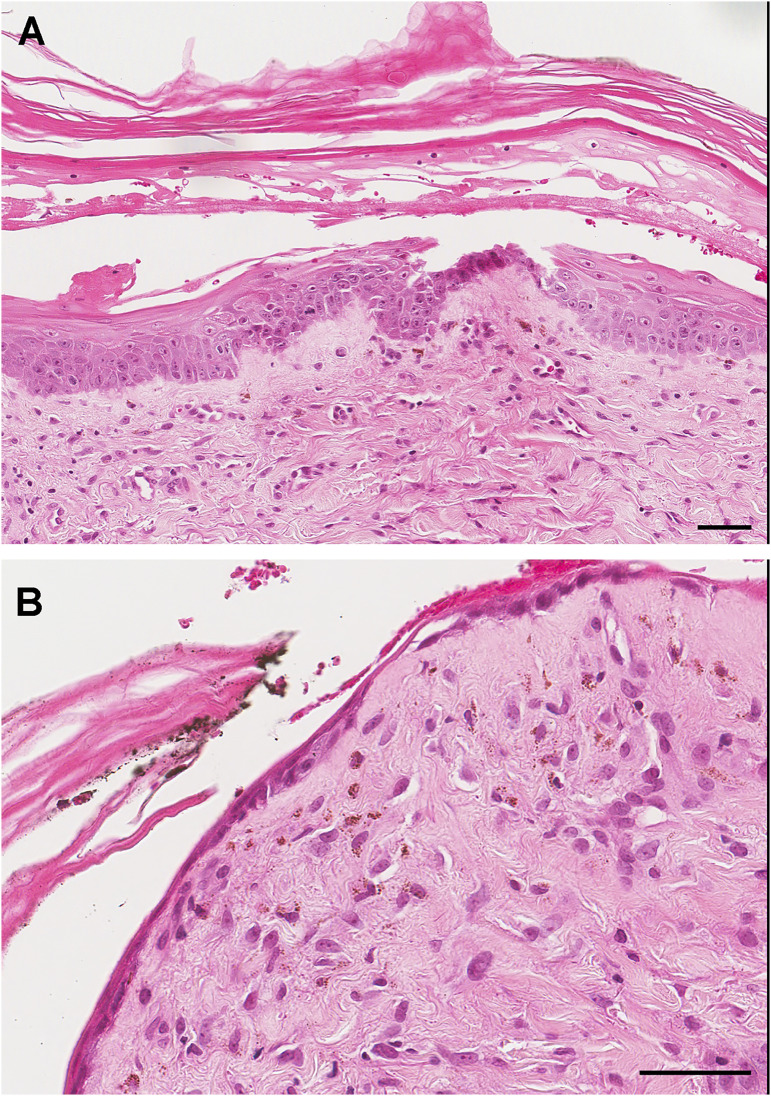
Skin biopsy demonstrates keratinolysis, bullae formation, separation of the overlying keratin layer and superficial dermal inflammatory infiltrate with few lymphocytes, macrophages and melanophages (A). The overlying epidermis was markedly denuded with no changes to the superficial dermis (B). Scale bar = 50 micrometers.

Chimerism testing using short tandem repeat analysis (multiplex PCR amplification of amelogenin locus and 15 microsatellite markers using AmpFLSTR Identifier PCR Amplification Kit, ThermoFischer Scientific) detected 99.6% liver donor DNA and 0.4% recipient DNA in both whole blood and myeloid cells (defined as positively selected CD33 + and CD66b + cells) and 7.3% liver donor DNA and 92.7% recipient DNA in skin. Although CD3 + T cells and CD19 + B cells appeared to be 100% donor-related, conclusive results were not obtained initially given the low number of these cells in blood samples. Samples were re-analyzed for CD3 + and CD19 + , with optimization for low DNA concentrations via polymerase chain reaction adjustment. This showed 97.5% donor CD3 + T cells and 98.8% CD19 + donor B cells, confirming the presence of donor lymphocytes in the blood, supporting a diagnosis of GVHD. A punch biopsy of the right leg rash demonstrated bullae formation, keratinolysis and denudation of the overlying keratin layer from the superficial epidermis, as well as necrosis. There was an associated superficial perivascular dermatitis (Figure 1). Although these findings are nonspecific, interpretation in the context of the clinical history and chimerism supports the diagnosis of GVHD, Grade IV (Skin Stage 4, Liver Stage 3, Gut Stage 4).

The patient started granulocyte colony-stimulating factor (filgrastim) 300 mcg subcutaneous daily, methylprednisolone 2 mg/kg/day intravenous daily and anti-thymocyte globulin (rabbit) 4.5 mg/kg total dose intravenous daily for three days. He received basiliximab 40 mg intravenous with four planned repeat weekly doses. The latter two agents were used due to the rapidly deteriorating condition of the patient. Unfortunately, the patient continued to decline, likely due to his GvHD, despite aggressive treatment. Due to his declining condition, the family elected to change his goals of care to focus on palliation with his ultimate demise later that day after the withdrawal of life support (Day 73 post-transplant).

## Discussion

Acute GVHD is mostly described after HSCT and generally presents two to six weeks following transplantation.^1^ Fever is often an early sign, followed by skin rash and diarrhea.^1^ Other gastrointestinal manifestations include abdominal pain, nausea, vomiting, anorexia and bleeding. Liver dysfunction is common and characterized by elevated bilirubin and alkaline phosphatase.^2^ Multilineage cytopenias or pancytopenia may indicate bone marrow dysfunction.

GVHD can also occur as a rare complication of organ transplantation and is characterized by high mortality. The incidence of GVHD varies with the type of transplant and is more common with small bowel (5.6%) and liver (0.5–2%) transplants.^1,3^ GVHD may be acute or chronic based on the onset of symptoms before or after an arbitrary duration of 100 days. Chronic GVHD may develop following acute GVHD or as de novo disease.

Liver transplant-associated GVHD occurs via two mechanisms. The first and more common process is a mild antibody-mediated hemolytic reaction that occurs when hosts with A, B or AB blood types receive solid organ grafts from donors with O blood type.^2^ The second form - discussed here - occurs when donor-derived immunocompetent T cells recognize host antigens of histo-incompatible cells in the skin, liver, gastrointestinal tract and bone marrow as foreign and mount an immune response.^1,2^ Passenger lymphocytes from transplanted organs may engraft and multiply in the host bone marrow. This leads to an immune-mediated attack on hematopoietic stem cells, resulting in bone marrow aplasia, pancytopenia and immunodeficiency.^1,3^ Replacement of recipient hematopoiesis through engraftment of passenger hematopoietic stem cells has also been reported.^4^

**Figure 2.**
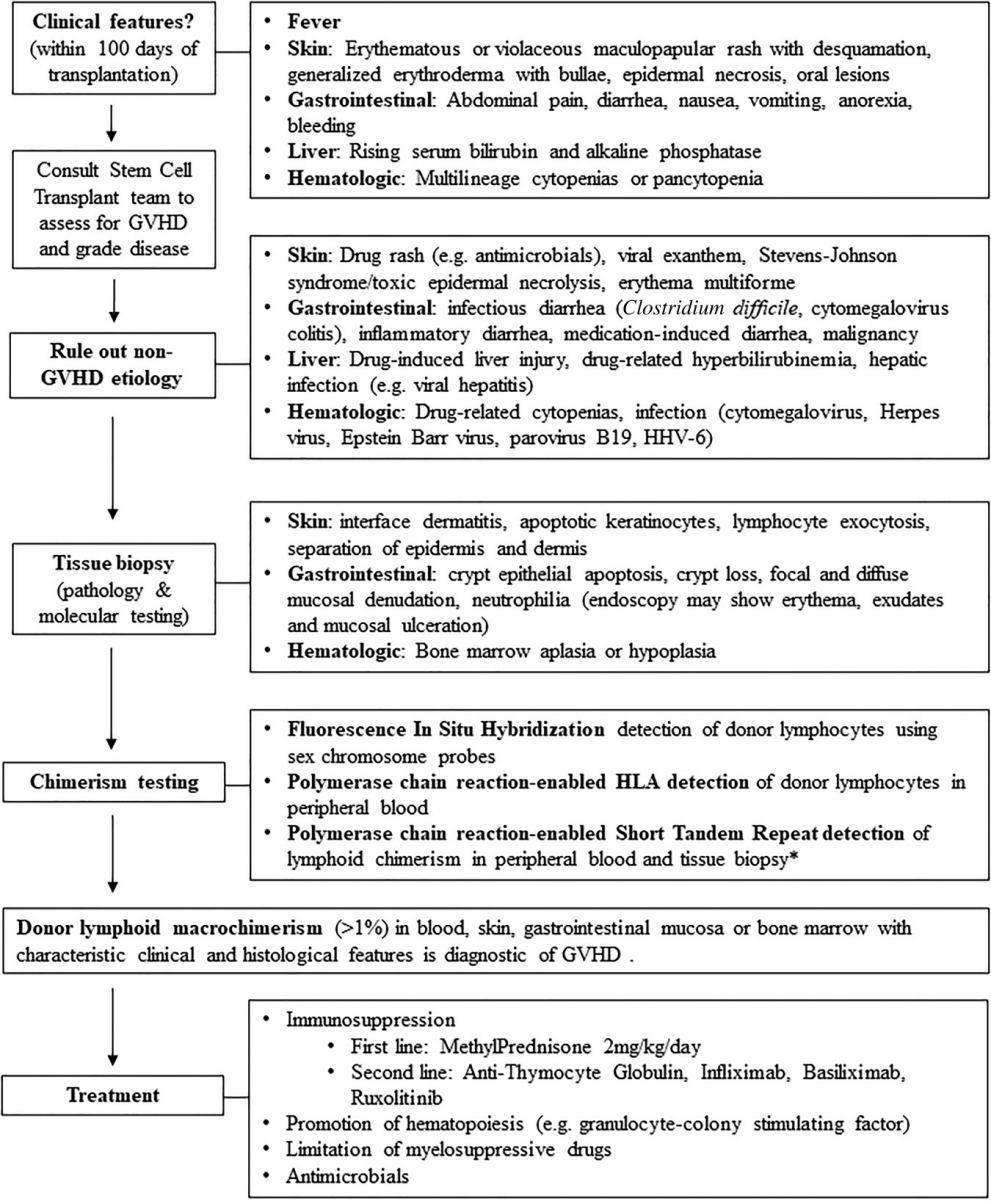
Selected considerations in the workup of suspected acute solid organ transplant-associated graft-versus-host-disease (SOT-GVHD).

GVHD can be challenging to diagnose as non-specific signs and symptoms may be attributed to comorbidities.^1^ We propose an algorithm for the workup of suspected acute GVHD (Figure 2). Non-GVHD etiologies must be ruled out including infection (e.g. *Clostridium difficile* and cytomegalovirus colitis), drug rash, drug-induced liver injury and drug or viral-related cytopenias (such as CMV, HHV-6). The diagnosis of GVHD for our patient was delayed because his symptoms were initially attributed to cytomegalovirus infection and ganciclovir, highlighting that the initial presentation of GVHD is often nonspecific. When GVHD was considered, it was initially suggested to be attributed to blood transfusion-associated GVHD. However, on review, past transfusions had been stored for over 14 days which significantly reduces the risk of transfusion-associated GVHD, due to the low viability of donor T-cells.

When GVHD is considered, we suggest consulting the HSCT team to help make the diagnosis and initiate treatment of the disease. In our case, pre-existing partnerships among the liver and HSCT teams, transfusion medicine specialists, and critical care specialists facilitated timely communication relevant to confirming GVHD. Acute GVHD is staged according to clinical manifestations with overall grades I-IV. Organ stages are classified from 0–4 using percent body surface area of skin involvement, total bilirubin levels and gut stool volume.^5^ Staging has prognostic value, but may be confounded by clinical subjectivity, interobserver variability and comorbidities. Our patient had overall Grade IV acute GVHD (Skin Stage 4, Liver Stage 3, Gut Stage 4) based on the modified Seattle Glucksberg/Przepiorka Criteria.

GVHD is diagnosed clinically but may be supported by the presence of donor lymphoid chimerism in blood, skin, gastrointestinal mucosa or bone marrow with characteristic clinical and histological features.^1,3,6^ Pathology of skin, gastrointestinal tissue and bone marrow may support the diagnosis. In isolation, biopsy findings are nonspecific, but the presence of apoptotic bodies and a vacuolar interface dermatitis may favour GVHD.^2,3^ Chimerism is confirmed with fluorescence in situ hybridization analysis (in case of mismatched sex transplants) of tissue to detect donor lymphocytes, polymerase chain reaction-enabled human leukocyte antigen detection of donor lymphocytes in peripheral blood, as well as polymerase chain reaction-enabled short tandem repeat detection of donor lymphocytes in peripheral blood and tissue biopsy.^6^ Donor lymphoid chimerism greater than 20% one-week post-transplant is specific for GVHD, although this value can be lower.^6^ Donor lymphoid macrochimerism (>1% of donor lymphocytes in host tissues) in a patient with clinical and histological findings is diagnostic of GVHD.^3^ This contrasts with microchimerism (<1%), which is often present in liver transplant recipients and may play a role in host acceptance of the graft.^3^ In our case, confirmation of GVHD was delayed because blood samples had DNA concentrations below the default detection threshold; chimerism for CD3 + T cells and CD19 + B cells was confirmed only after validated modification of the detection process.

Mortality from GVHD is high - approximately 75–85% in liver transplants, 100% in lung transplants and 30% in other solid organ transplants.^3,6^ Most patients die from overwhelming infection, multiorgan failure or hemorrhage secondary to bone marrow dysfunction.^3,6^ Studies on predictors of mortality from GVHD in the context of liver transplantation highlight age differences between donor and recipient, lag time between presentation and diagnosis, HLA- matches between donor and recipient, as well as the presence of pancytopenia or diarrhea as key predictors of poor outcome.^7,8^ GVHD after organ transplantation lacks guideline-directed treatment. Therapy includes immunosuppressants, promoting hematopoiesis with recombinant cytokines and limiting myelosuppressive drugs.^6^ Treatment is more effective if started before the patient develops pancytopenia. Standard initial treatment is high-dose corticosteroids. Case reports suggest benefits with interleukin-2 antagonists (i.e. basiliximab), tumour necrosis factor alpha inhibitors (i.e. infliximab and etanercept), alefacept (a since-discontinued fusion protein inhibiting CD4 and CD8 T lymphocytes), anti-thymocyte globulin, and anti-lymphocyte globulin.^2,3^ Broad-spectrum antimicrobial and antiviral agents are important adjuncts to prevent infectious complications. We are unaware of any international registries documenting cases of liver transplant-associated GVHD but assert that systematic surveillance could improve the dissemination of information on treatments and outcomes to clinicians. To summarize, our case highlights the challenges of diagnosing acute GVHD after liver transplantation, and the value of involving the HSCT team to facilitate prompt recognition and treatment. We argue that early involvement of the HSCT team in the diagnosis and management of SOT-GVHD is critical for efficient delivery and timely care to these patients.
